# Inhibitory Activities of Cyanidin and Its Glycosides and Synergistic Effect with Acarbose against Intestinal α-Glucosidase and Pancreatic α-Amylase

**DOI:** 10.3390/ijms11093387

**Published:** 2010-09-20

**Authors:** Sarinya Akkarachiyasit, Piyawan Charoenlertkul, Sirintorn Yibchok-anun, Sirichai Adisakwattana

**Affiliations:** 1 Department of Pharmacology, Faculty of Veterinary Science, Chulalongkorn University, Bangkok 10330, Thailand; E-Mails: akkara_sarin@hotmail.com (S.A.); piyawanch@yahoo.com (P.C.); sirintorn.y@chula.ac.th (S.Y.); 2 The Medical Food Research and Development Center, Department of Transfusion Medicine, Faculty of Allied Health Sciences, Chulalongkorn University, Bangkok 10330, Thailand

**Keywords:** cyanidin glycosides, intestinal α-glucosidase, pancreatic α-amylase, synergism

## Abstract

Cyanidin and its glycosides are naturally dietary pigments which have been indicated as promising candidates to have potential benefits to humans, especially in the prevention and treatment of diabetes mellitus. We investigated the structure activity relationships of cyanidin and its glycosides to inhibit intestinal α-glucosidases and pancreatic α-amylase *in vitro.* The results found that cyanidin and its glycosides are more specific inhibitors of intestinal sucrase than intestinal maltase. Cyanidin-3-galactoside and cyanidin-3-glucoside were the most potent inhibitors against intestinal sucrase and pancreatic α-amylase with IC_50_ values of 0.50 ± 0.05 and 0.30 ± 0.01 mM, respectively. Our findings indicate that the structural difference between glucose and galactose at the 3-*O*-position of cyanidin was an important factor for modulating the inhibition of intestinal sucrase and pancreatic α-amylase. The combination of cyandin-3-glucoside, cyanidin-3- galactoside or cyanidin-3,5-diglucosides with a low concentration of acarbose showed synergistic inhibition on intestinal maltase and sucrase. The synergistic inhibition was also found for a combination of cyanidin or cyanidin-3-glucoside with a low concentration of acarbose. The findings could provide a new insight into a use for the naturally occurring intestinal α-glucosidase and pancreatic α-amylase inhibitors for the prevention and treatment of diabetes and its complications.

## 1. Introduction

Anthocyanins are the largest group of water-soluble pigments in the Plant Kingdom. They have been recently demonstrated to have potential health benefits and disease prevention properties in animals and humans. Anthocyanins are included in the list of natural compounds known as potential antioxidants [1]. Consumption of anthocyanin-enriched foods is associated with a reduced risk of several diseases such as atherosclerosis [2] dyslipidemia [3], and diabetes [4]. The delaying digestion of disaccharides to absorbable monosaccharides by inhibition of α-amylase and α-glucosidase is one therapeutic approach for controlling postprandial hyperglycemia in pre-diabetes and diabetes [5]. The suppression of postprandial hyperglycemia subsequently delays the progression of micro- and macro-vascular complications such as microangiopathy, cardiovascular, and cerebrovascular diseases [6].

**Figure 1:**
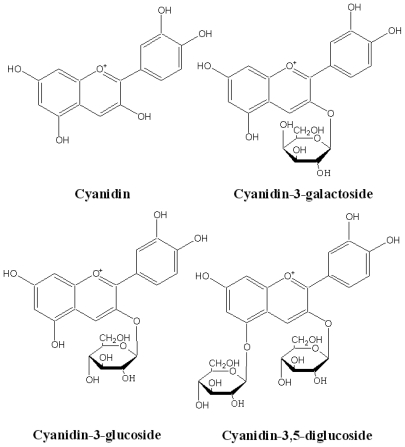
The structure of cyanidin and its glycosides.

Cyanidin and its glycosides (Figure 1) belong to anthocyanins, and are widely distributed in various human diets through crops, vegetables, fruits, and red wine suggesting that we daily ingest significant amounts of these compounds from plant-based diets [7]. Interestingly, cyanidin and its glycosides represent one of the major groups of naturally occurring anthocyanins; their mechanisms related to anti-diabetic effects have been deeply investigated [4]. Recent studies suggesting that cyanidin-3- galactoside potentially inhibits mammalian intestinal α-glucosidases have renewed interest in studies on delaying postprandial hyperglycemia and hyperinsulinemia. The kinetic model proposed for this type of inhibition shows that cyanidin-3-galactoside is a mixed type pattern against intestinal sucrase [8]. The combination of acarbose and cyanidin-3-galactoside has a synergistic effect on intestinal α-glucosidase inhibition [8]. Our previous study indicates that cyanidin-3-rutinoside also exhibits an inhibitory effect on yeast α-glucosidase in a non-competitive manner [9]. Although the α-glucosidase inhibitory activities of cyanidins are well-documented, studies regarding the structureactivity relationship of cyanidin and its glycosides on the inhibition of α-glucosidases and pancreatic α-amylase have not been undertaken. Therefore, the aim of this study was to investigate the structure relationship of cyanidin and its glycosides on inhibition of mammalian α-glucosidases and pancreatic α-amylase. Furthermore, the combined effect of acarbose by these compounds was also investigated. The results of our investigation provide useful information for the development of natural compounds in order to prevent and treat diabetes mellitus.

## 2. Material and Methods

### 2.1. Chemicals

Rat intestinal acetone powder, porcine pancreatic α-amylase, 3,5-dinitrosalicylic acid, glucose oxidase kits were purchased from Sigma Chemical Co. Ltd. (St. Louis, MO). Acarbose was obtained from Bayer, Germany. Cyanidin chloride, cyanidin-3-glucoside chloride, cyanidin-3-galactoside chloride, and cyanidin-3,5-diglucoside were purchased from Chromadex, USA. All others chemicals used were of analytical grade.

### 2.2. α-Glucosidase Inhibition Assay

The assessment of α-glucosidase inhibitory activity was performed according to our previous report [10]. Briefly, 100 mg of rat intestinal acetone powder was homogenized in 3 mL of 0.9% NaCl solution. After centrifugation (12,000 g × 30 min), 10 μL of the supernatant (maltase = 0.68 units/mg protein, sucrase = 0.10 units/mg protein) was incubated with 70 μL of substrate solution (37 mM maltose, 54 mM sucrose), and 20 μL of sample at various concentrations in 0.1 M phosphate buffer pH 6.9 at 37 °C for 30 min (maltase assay) and 60 min (sucrase assay). The mixtures were suspended in boiling water for 10 min to stop the reaction. The concentrations of glucose released from the reaction mixtures were determined by glucose oxidase method. Acarbose was used as a positive control for this assay.

### 2.3. Pancreatic α-Amylase Inhibition Assay

The pancreatic α-amylase inhibition assay was performed according to our previous reports [10]. Porcine pancreatic α-amylase was dissolved in 0.1 M phosphate buffer saline, pH 6.9. The various concentrations of test compounds were added to solution containing in 1 g/L starch and phosphate buffer. The reaction was initiated by adding amylase (3 U/mL) to the incubation medium to a final volume of 500 μL. After 10 min, the reaction was stopped by adding 0.5 mL dinitrosalicylic (DNS) reagent (1% 3,5-dinitrosalicylic acid, 0.2% phenol, 0.05% Na_2_SO_3_, and 1% NaOH in aqueous solution) to the reaction mixture. The mixtures were heated at 100 °C for 10 min and 500 μL of 40% potassium sodium tartarate solution was added to the mixtures to stabilize the color. After cooling to room temperature in a cold water bath, absorbance was recorded at 540 nm using a spectrophotometer. Acarbose was used as a positive control for this assay

### 2.4. Combined Effect of Acarbose by Cyanidin and Its Glycosides

The various concentrations of acarbose were combined with or without cyanidin and its glycosides at low concentration. The reaction was performed according to the above assay (α-glucosidase and pancreatic α-amylase). Results were expressed as a percentage inhibition of the corresponding control values.

### 2.5. Statistical Analysis

Data were expressed as means ± S.E.M. The IC_50_ values were calculated from plots of log concentration of inhibitor concentration *versus* percentage inhibition curves by using Sigma Plot 10.0 (IL, USA). Statistical analysis was performed by Student’s t test.

## 3. Results

As shown in Table 1, cyanidin and its glycosides moderately inhibited intestinal sucrase but were not active against intestinal maltase. The IC_50_ value of cyanidin was 1.42 ± 0.25 mM against intestinal sucrase. Upon the introduction of 3-*O*-glucose moiety of cyanidin, it was found that the IC_50_ value of cyanidin-3-glucoside decreased intestinal sucrase inhibition by 1.46-fold as compared with cyanidin. Furthermore, the addition of 5-*O*-glucose moiety in cyanidin-3-glucoside gave cyandin-3,5- diglucoside, which is an inactive intestinal sucrase inhibitor. Meanwhile, replacement of a residue at 3-*O*-glucose of cyanidin by a 3-*O*-galactose moiety resulted in a significant increase in intestinal sucrase inhibitory activity. It was observed that intestinal sucrase inhibitory activities increased in the order of cyanidin-3-galactoside > cyanidin-3-glucoside > cyanidin > cyanidin-3,5-diglucoside. However, cyanidin and its glycosides were less potent than that of acarbose on the intestinal maltase and sucrase inhibition.

Table 1 shows the ability of each compound to inhibit pancreatic α-amylase. The IC_50_ value of cyanidin-3-glucoside was a better pancreatic α-amylase inhibitor than the other three kinds of cyanidins. Meanwhile, cyanidin-3-galactoside and cyanidin-3,5-diglucoside had no inhibitory activity against pancreatic α-amylase. It should be noted that acarbose with pancreatic α-amylase inhibitory activity, which was used as a positive control, showed an IC_50_ of 0.12 ± 0.04 mM in our assay system.

**Figure 2:**
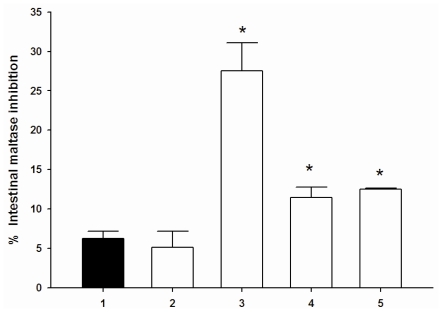
The combined effect of acabose and cyanidins on intestinal maltase inhibition. (**1**): 0.05 μM acarbose; (**2**): 0.05 μM acarbose + 1 μM cyanidin; (**3**): 0.05 μM acarbose + 1 μM cyanidin-3-glucoside; (**4**): 0.05 μM acarbose + 1 μM cyanidin-3-galactoside; (**5**): 0.05 μM acarbose + 1 μM cyanidin-3,5-diglucoside. Result are expressed as means ± S.E.M; *n* = 3. *P < 0.001 compared with acarbose (0.05 μM) alone.

It was of interest to establish whether cyanidin and its glycosides and acarbose might interact synergistically on intestinal α-glucosidase and pancreatic α-amylase. Therefore, the assay was then performed in solutions containing acarbose alone or in mixture with a low concentration of these compounds (1 μM). The combined effects of acarbose together with cyanidin and its glycosides on intestinal maltase inhibition are shown in Figure 2.

**Figure 3:**
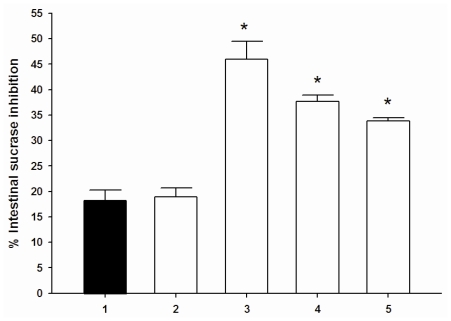
The combined effect of acabose and cyanidins on intestinal sucrase inhibition. (**1**): 3.12 μM acarbose; (**2**): 3.12 μM acarbose + 1 μM cyanidin; (**3**): 3.12 μM acarbose + 1 μM cyanidin-3-glucoside; (**4**): 3.12 μM acarbose + 1 μM cyanidin-3-galactoside; (**5**): 3.12 μM acarbose + 1 μM cyanidin-3,5-diglucoside. Result are expressed as means ± S.E.M; *n* = 3. *P < 0.001 compared with acarbose (3.12 μM) alone.

The results showed that cyanidin and its glycosides (1 μM) had no inhibitory activity on intestinal maltase and sucrase (data not shown). When cyanidin-3-galactoside, cyanidin-3-glucoside, and cyanidin-3,5-diglucoside was added to the assay system with acarbose (0.05 μM), the percentage intestinal maltase inhibition was increased when compared with acarbose alone. When each compound was added to the assay system containing a low concentration of acarbose (3.12 μM), the percentage intestinal sucrase inhibition markedly increased (Figure 3). No change in the percentage of intestinal maltase and sucrase inhibition was observed in the presence of the combination of low concentration of cyanidin and acarbose. The findings indicate that cyanidin-3-galactoside, cyanidin-3-glucoside, and cyanidin-3,5-diglucoside produce synergistic effects on intestinal maltase and sucrase inhibition when combined with a low concentration of acarbose.

**Figure 4:**
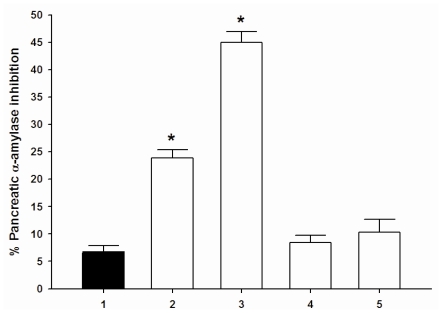
The combined effect of acabose and cyanidins on pancreatic α-amylase inhibition. (**1**): 3.12 μM acarbose; (**2**): 3.12 μM acarbose + 1 μM cyanidin; (**3**): 3.12 μM acarbose + 1 μM cyanidin-3-glucoside; (**4**): 3.12 μM acarbose + 1 μM cyanidin-3-galactoside; (**5**): 3.12 μM acarbose + 1 μM cyanidin-3,5-diglucoside. Result are expressed as means ± S.E.M; *n*= 3. *P < 0.001 compared with acarbose (3.12 μM) alone.

The results showed that cyanidin and its glycosides (1.0 μM) had no inhibitory activity on pancreatic α-amylase inhibition (data not shown). When adding each compound (1.0 μM) to acarbose (3.12 μM), cyanidin or cyanidin-3-glucose significantly increased the percentage pancreatic α-amylase inhibition (Figure 4), whereas cyanidin-3-galactoside and cyanidin-3,5-diglucoside did not show significant changes in the percentage inhibition when compared acarbose alone. Our findings suggest that cyanidin and cyanidin-3-glucoside produce synergistic effect on pancreatic α-amylase inhibition when combined with a low concentration of acarbose.

## 4. Discussion

This is the first study to investigate the structure-activity relationships of cyanidin and its glycosides on intestinal α-glucosidase (maltase and sucrase) and pancreatic α-amylase inhibition. According to our results, inhibition of intestinal sucrase by cyanidin and its glycosides is more specific than inhibition of intestinal maltase. In addition, cyanidin is a weak pancreatic α-amylase and intestinal sucrase inhibitor, whereas cyanidin-3-glucoside is a more potent inhibitor than cyanidin. These results indicate that the presence of 3-*O*-glucoside is important for inducing potent inhibition against pancreatic α-amylase and intestinal sucrase. Increasing bulkiness at the 5-*O*-position by the glucose moiety of cyanidin-3-glucoside dramatically decreased the potency of pancreatic α-amylase and intestinal sucrase inhibition, suggesting that this certain position may not necessarily require the presence of a glucose residue. This position may serve as the region for binding to the active site of pancreatic α-amylase and intestinal sucrase.

One interesting finding is that the replacement of 3-*O*-glucose of cyanidin-3-glucoside by a galactose residue directly increases intestinal sucrase inhibitory activity, whereas it dramatically decreases pancreatic α-amylase inhibitory activity. When comparing the structure of glucose and galactose, the molecules have the same molecular formula but different structural formulae. The position of the hydroxyl (-OH) group on C-4 is the only distinction between glucose and galactose (Figure 1). We suggest that the structural difference of the sugar at the 3-*O*-position may be an important factor for modulating the inhibition of intestinal sucrase and pancreatic α-amylase. A recent study has shown that the inhibitory activity of flavonols and flavones depends on hydrogen bonds between the hydroxyl groups of the polyphenol ligands and the catalytic residues of the binding site and formation of a conjugated pi-system that stabilizes the interaction with the active site [11]. The molecular interaction of cyanidin and its glycosides on specific binding site on intestinal α-glucosidase and pancreatic α-amylase remains unclear. From the information of flavonols and flavones mentioned above, it can be assumed that cyanidin and its glycosides may interact with protein by using hydroxyl groups in their molecular structure, resulting in the formation of hydrogen bonds with the polar groups (amide, guanidine, peptide, amino and carboxyl groups) in the active site of proteins by covalent and/or non-covalent interactions. To prove this hypothesis, X-ray crystallography and computer modeling to evaluate the binding activity of cyandin and its glycosides on intestinal α-glucosidase and pancreatic α-amylase is needed to further investigation.

Cyanidin-3-glucoside is a typical anthocyanin that is most abundant in black rice and purple corn [12,13]. It has been shown that dietary cyanidin-3-glucoside-riched purple corn significantly reduces blood glucose concentration and enhances insulin sensitivity in type 2 diabetic mice by upregulating the glucose transporter 4 (Glut4) and downregulating retinol binding protein 4 (RBP4) in the white adipose tissue [14]. In addition, cyanidin-3-glucoside also demonstrates a protective action against H_2_O_2_- or TNF-α-induced insulin resistance in 3T3-L1 adipocytes by inhibiting the c-Jun NH_2_-terminal kinase (JNK) signal pathway [15]. Recent studies have shown that cyanidin-3-glucoside and cyanidin-3-galactoside directly stimulate insulin secretion from a pancreatic β-cell line [16]. Considering the data obtained from our investigations, it is especially noteworthy that cyanidin and its glycosides play another role in controlling of postprandial hyperglycemia by inhibiting intestinal α-glucosidase and pancreatic α-amylase. This inhibitory action may contribute to decreasing the level of HbA_1c_, resulting in a significant reduction in the incidence of chronic vascular complication in diabetic patients [17].

Acarbose, α-glucosidase inhibitor, has been clinically studied in type 2 diabetes mellitus. A recent report has shown that acarbose treatment was associated with a 25% reduction in the incidence of diabetes [18]. Treatment of acarbose leads to a 20% reduction of the postprandial peak of glycemia. This effect may last for as much as 5 h, with an increase in the time of glucose absorption that prevents glucotoxicity and the consequent hyperinsulinemia [19]. The lowest dose of acarbose with clinical effects is 150 mg/day, with doses >300 mg/day already exceeding the saturated binding of α-glucosidase, and causing no increase in the inhibitory effect of this drug [20,21]. The most common adverse effect of acarbose is gastrointestinal disturbance such as flatulence, meteorism and abdominal distention which occurs in a dose-dependent manner [19]. Anthocyanins occur frequently in food items such as berries, vegetables, cereals, and red wines. The average of daily intake of anthocyanins in human has been estimated to be 180–215 mg [22]. Thus, it is possible that intake of cyanidin and glycosides-enriched plant foods together with acarbose may lead to the development a novel combined therapy in type 2 diabetic patients. Our present study shows that combination with a low concentration of acarbose and some cyanidins produces more synergistic inhibition than either drug alone, suggesting that they may provide a significant clinical benefit in delaying postprandial hyperglycemia. As a consequence of these synergistic effects, it is possible to reduce dosage of acarbose by combination with cyanidin and its glycosides, resulting in a diminished adverse effect of acarbose. The intake amount of cyanidin and its glycosides that is required together with low dose of acarbose also needs further investigation in diabetic patients.

## 5. Conclusion

Although cyanidin and its glycosides have been investigated for possible mechanisms of anti-diabetic activities, the present study shows that these compounds markedly inhibit intestinal α-glucosidase and pancreatic α-amylase, which is one of the therapeutic approaches for treatment of diabetes mellitus. This is the first report of the combined effect of acarbose and cyanidin and its glucosides. Improvement in postprandial hyperglycemia, hyperinsulinemia and insulin resistance, treatment of an overweight condition and diminishing of the adverse effects of acarbose in diabetic control can be clinically achieved by this combination.

## Figures and Tables

**Table 1:** The IC_50_ values for intestinal α-glucosidase (maltase and sucrase) and pancreatic α-amylase by cyanidin and its glucosides.

Compounds	IC_50_ values (mM)		
	Maltase	Sucrase	α-Amylase
Cyanidin	>3.00	1.42 ± 0.25	0.38 ± 0.01
Cyanidin-3-glucoside	>3.00	0.97 ± 0.05	0.30 ± 0.01
Cyanidin-3-galactoside	>3.00	0.50 ± 0.05^a^	>1.00
Cyanidin-3,5-diglucoside	>3.00	>2.00	>1.00
Acarbose	0.003 ± 0.001	0.09 ± 0.04	0.12 ± 0.04

Results were expressed as means ± S.E.M., n = 3.

a The IC_50_ value of cyanidin-3-galactoside was previously reported in Adisakwattana *et al.* [8].
